# Does ‘COVID-19 phobia’ stimulate career anxiety?: Experience from a developing country

**DOI:** 10.1016/j.heliyon.2021.e06346

**Published:** 2021-03-08

**Authors:** Md. Shahed Mahmud, Md. Mahbubar Rahman, Md. Masud-Ul-Hasan, Md. Azharul Islam

**Affiliations:** a Department of Management, Mawlana Bhashani Science and Technology University, Tangail, Bangladesh; b Department of Marketing, University of Rajshahi, Rajshahi, Bangladesh; c Department of Marketing, Begum Rokeya University, Rangpur, Bangladesh; d Department of Textile Engineering, Mawlana Bhashani Science and Technology University, Tangail, Bangladesh

**Keywords:** COVID-19 phobia, Specific phobia, General anxiety disorder, Career anxiety, PLS-SEM

## Abstract

Due to the outbreak of COVID-19 different forms of anxiety disorder have been seen in the mindset of people all around the world. This study aims to examine a structural relationship between ‘COVID-19 phobia’ and career anxiety among the workforce from the perspective of a developing country. This study collected survey data using a structured questionnaire by applying the scales of ‘COVID-19 Phobia’ and career anxiety. Study results reveal that the factors of the ‘COVID-19 phobia’ have a substantial influence on generating career-related anxiety among the workforce. Study results can play a vital role for the policymakers to formulate long-term policies to retrieve the world's economy. This study combined the concept of specific phobia and general anxiety disorder (GAD) to figure out how the global pandemic impacted peoples' mindsets and create career anxiety. The study results have theoretical and practical implications in many folds.

## 1 Introduction

Outbroken from Wuhan, China, in late 2019, novel coronavirus is spreading like wildfire, showing no signs of halt and makes a grim situation throughout the world (WHO, 2020b). Over the last centuries, COVID-19 like pandemics emerged in different parts of the world and created havoc for the humankind (Taylor, 2019). Till now, among the affected people, patients relatively older ages and having different chronic diseases, are the worst sufferer in the death toll count (Gates, 2020; Li et al., 2020; Velavan and Meyer, 2020). The world is witnessing a brand new epidemiological as well as a psychological catastrophe, with the spread of the COVID-19 pandemic (Mukhtar, 2020; Torales et al., 2020). Like the predecessors of the COVID-19 pandemic, human mental health going through an unimaginable critical situation (Balaratnasingam and Janca, 2006; Torales et al., 2020). Different psychological distresses like, anxiety, trauma, depression, anger, panic, and some other mental illness are some of the common mental illnesses widely seen in pandemic situations (Brooks et al., 2020; Islam et al., 2020; Moghanibashi-Mansourieh, 2020; Taylor et al., 2008; Tucci et al., 2017; Vibha et al., 2020; WHO, 2020a). The current pandemic fosters economic uncertainty and creates a new form of crisis all over the world.

The long-lasting effect of the COVID-19 trimming world's economy in an unprecedented manner (Bakker and Wagner, 2020). Industries, all around the world, are thriving and leaders all around the world are taking severe measures for the survival of the economy. Global demand for products has dropped (i.e., first time in the history of the world, the price of oil in the USA gone negative) and a potential great financial depression is threatening the world's economy after the 1930s (Goodman, 2020; Oxford Economics, 2020; Riley, 2020; Sheppard et al., 2020). Companies all around the world are adopting different cost minimization strategies (i.e., reducing the number of employees or cutting the existing salaries of the employees, etc.) for the existence (Coibion et al., 2020; Sarkar, 2020). Recent reports of the International Monetary Fund (IMF) and International Labor Organization (ILO) predict that the forthcoming financial recession will permanently damage the world's economic system and the unemployment rate will be in a record high (BBC, 2020b; ILO, 2020a; Kawohl and Nordt, 2020; Martin & BLOOMBERG, 2020). With the presence of fear and uncertainty, the people working in different sectors and potential employees are facing numerous mental distress (Bao et al., 2020; Charles and Anderson-Nathe, 2020). Different forms of formal and informal media also boost-up the whole process of a mental burden sometimes by providing false and unwanted information (WHO Director-General leveled the trend as “Infodemic”) (Bora et al., 2018; Shuja et al., 2020; The Lancet, 2020). Thus, the spread of the pandemic creating a heavy psychological burden in all folks of people, especially among the younger working class. ILO labeled these younger peoples as a ‘lockdown generation’ and predicted that a vast chank of employed people would lose their current job and face a multi-dimensional shock and thus the suicidal case will go up especially among the ‘lockdown generation’ (Goyal et al., 2020; ILO, 2020a; 2020b; Kawohl and Nordt, 2020; Killgore et al., 2020; Mamun and Griffiths, 2020). People tend to scare about the spread of the virus and unable to concentrate on their regular work and cause severe damages. For instance, the initial report on a recent plane crash in Pakistan reveals that, the operating pilots are talking about coronavirus and done a human mistake resulting 97 deaths (BBC, 2020c).

Clinical results found that the spread of infectious diseases can create anxiety disorder on human psychology (Taylor, 2019) and COVID-19, also creating anxiety disorder in the mindset of humans all around the world (Chakraborty, 2020). Asmundson and Taylor (2020) termed this phenomenon as “coronaphobia” and argued that this new breed of phobia has an immense impact on human psychology. As the world's economy is shrinking, the workforce (current and future) all around the world are getting anxious about their career. On this backdrop, this study aims to examine the relationship of ‘COVID-19 Phobia’ with career anxiety. Due to the long-lasting and deadly nature, COVID-19 making some forms of phobia among people all around the world. Again, the working class (existing and potential) are facing career related anxiety and concern about their future. Based on this assumption, this study hypothesized a structural relationship by adopting and applying ‘COVID-19 Phobia’ and career anxiety-related scales. The remaining article organized as follows-firstly the theoretical background is presented followed by measurement scales and hypotheses. The next sections include methodology, analysis and discussion, and finally, the article concludes with conclusion and implications, and limitations and future research direction.

## 2 Theoretical background

Anxiety is the emotional and physiological response of human anticipation of future threats (APA, 2017). Though anxiety is a common phenomenon for human psychology, an excessive amount of anxiety creates anxiety disorder, which is harmful to human health (APA, 2017; Holland, 2018). Different types of anxiety disorders like Specific Phobia, Social Anxiety Disorder, Panic Disorder, Agoraphobia, Generalized Anxiety Disorder (GAD), etc. are some of the common anxiety disorders found in human psychology (APA, 2017). Human psychology is suffering from different forms of anxiety-related issues and among them, specific phobia and GAD playing a crucial role because of the outbreak of COVID-19 (Torales et al., 2020).

Specific phobia is *“an anxiety disorder, formerly called simple phobia, characterized by a marked and persistent fear of a specific object, activity, or situation”* (American Psychological Association, 2020). According to Black and Grant (2014), *“an individual with specific phobia becomes immediately frightened or anxious when presented with a feared object.”* Different physiological experiences or environmental conditions can play as a role of catalyst to generate specific phobia of humans (Black and Grant, 2014). In this perspective, any human-made or natural disaster having deadly effects can be considered under specific phobia (Arpaci et al., 2020). Pandemics like COVID-19 spreading all around the world, the number of infected people and death tolls are increasing day by day, regular activities of the people extremely disrupted like no other pandemics in the past (Brooks et al., 2020; Tucci et al., 2017). Thus, affective and behavioral parts of human psychology are severely affected due to the outbreak of COVID-19 and this novel psychological distress can be listed as a specific phobia. According to Arpaci et al. (2020), *“specific phobias can trigger other anxiety disorders and are reported to comorbid with suicidal tendency, major depression, anxiety disorders, and physical, mental, or mood disorders.”*

Again, because of the spread of COVID-19, the regular financial condition and standard of living of people all around the world are harshly interrupted. It is becoming a crisis of human, economic and social at a time and attacked ‘societies at their core’ (United Nations (UN), 2020a). Thus, the regular mental health of humans is seriously hammered by the coronavirus anxiety and created a new breed of GAD under the anxiety disorder domain. GAD is *“an anxiety disorder characterized by excessive anxiety and worry across multiple content domains that is experienced as uncontrollable by the person”* (Portman et al., 2012). The worry sometimes got intensive on several content areas like job-related aspects, financial, health, and safety-related aspects of a person's oneself or family members (Portman et al., 2012). Previous researchers and different models and theories postulate that a person having GAD suffers from different chronic anxiety disorders and that influence their regular activities also (Portman et al., 2012). Because of the spread of coronavirus and its long-lasting nature (BBC, 2020a), the economy of the world has been hampered brutally and the organizations have to cut either salary or their regular workforce for their existence (Babu and Siddiqui, 2020; Bachman, 2020; Bhattarai et al., 2020; Davies, 2020; Kuttappan, 2020; Mukul, 2020; Schwartz, 2020). Thus, the people working in different sectors are in a fear of job loss or income reduction associated crisis, and this crisis trigger career-related anxiety (Mahmud et al., 2020). Hence, in this study, the authors proposed career anxiety as a worry about future career in terms of losing job, cut throat competition in getting new jobs and sustaining existing jobs and reducing the salary and so on under GAD.

## 3 Measurement scales and hypotheses

### 3.1 COVID-19 phobia scale (C19P–S)

On the background of global pandemic (COVID-19), a new scale called ‘COVID-19 Phobia Scale’ (C19P–S) was developed and validated by Arpaci et al. (2020), a four construct (Psychological factors, Social factors, Economic factors, Psycho-somatic factors) scale, specially designed for measuring the COVID-19 phobia (see Appendix 1) used in this study as independent variables.

### 3.2 Career anxiety scale

Current and future workforce form all around the world are getting feared for getting new jobs and retaining existing ones, because of the outbreak of COVID-19 pandemic. The fear of death and uncertainty of future livelihood escalates the fear in many folds (Mahmud et al., 2020). Previous scales related to job and career anxiety developed and validated by Tsai et al. (2017) and Schmalbach et al. (2020) are being considered here in this study for measuring career-related anxiety. For this study purpose, the career anxiety scale has been applied as a dependent variable.

**Figure 1:**
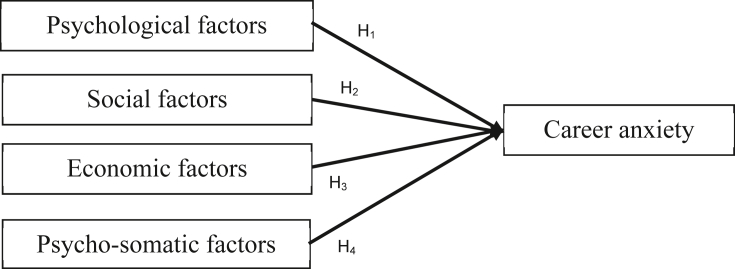
Research model.

Based on the constructs of COVID-19 phobia scales, the following hypotheses can be formed for this study (see Figure 1).

H1 Psychological factors have a substantial effect on developing career anxiety.

H2 Social factors have a significant impact on developing career anxiety.

H3 Economic factors have a significant impact on developing career anxiety.

H4 Psycho-somatic factors have a substantial effect on developing career anxiety.

## 4 Methodology

### 4.1 Survey design

The population for this study constitutes the persons who are currently seeking a job or working in different sectors all over the world. A purposive sampling technique was employed to select samples from the population for this study. To address the objective of the research, the persons who have at least completed their graduation, currently seeking a job or working in any job sector in Bangladesh, were being considered as a sample for this study. As this study applied PLS-SEM for empirical analysis thus, applying the ‘ten times rule of thumb’ (Hair et al., 2017) and statistical power analysis (Cohen, 1992), the minimum sample size was determined. This study was entirely carried out through an online-based questionnaire (google form). A step-by-step approach was followed for developing the structured questionnaire. At the very beginning, an extensive literature review was done to select measurement items, where Arpaci et al. (2020)'s ‘COVID-19 Phobia’ scale, Schmalbach et al. (2020)'s ‘Job-related worrying’ scale, and Tsai et al. (2017)'s ‘Employment Environment’ scale remained the base. Some relevant constructs were skimmed after the extensive literature review. Keeping the skimmed constructs into account, two idea-sharing sessions were conducted (through Zoom online platform) with fifteen jobseekers and private sector employees who are facing career-related anxiety because of the outbreak of COVID-19. Based on the summary of the discussions and the stated scales, finally five constructs were taken into consideration for conducting this study. With the assistance of language experts, the finalized English version questionnaire was translated into Bangla version. For the easy understanding of the questionnaire items the translation was done. Section-A and Section-B of the final questionnaire contains demographic information and questions regarding five constructs on the research topic respectively. Participants responded to all construct items in a five-point Likert scale. The 05-point scale ranged from 1 = strongly agree to 5 = strongly disagree.

### 4.2 Data collection and the ethics

An online-based survey questionnaire was served to the targeted population for data collection. Different social media platforms (i.e., Facebook Messenger, Linkedin, WhatsApp, Viber, etc.) were being used for the data collection. The data was collected from 05 June 2020 to 05 July 2020 time period. A total of 443 respondents' data was collected throughout the period. Finally, 432 (97.52 percent) responses were taken into consideration, from total of 443 samples, because they met all the required conditions and 11 responses had to drop as a result of straight-lining related problems (Kim et al., 2019). Helsinki's statement of research ethics was shadowed for this study. The online survey questionnaire contained the purpose and outcome of the study and also the rights of the respondents. All participants provided informed consent where the anonymity and confidentiality of the data were upheld strictly. Permission for rolling out the study was taken from the Department of Business Administration, Mawlana Bhashani Science and Technology University.

### 4.3 Data analysis

A PLS-SEM modeling approach was chosen to estimate structural equation models and to test the hypotheses. A variance-based SEM is chosen, as it well-fits the features and the nature of the data collected for this study (Hair et al., 2017). As the present study is exploratory in nature and employed a five-point Likert scale to collect primary data, so the non-normality of the distribution of data was assumed. Thus, PLS-SEM statistical analysis was applied as because this analysis can handle non-normal data and can handle relatively small sample size (Hair et al., 2017). For this study purpose, IBM SPSS (v23) and SmartPLS (v.3.2.9) software were applied to analyze data and testing the hypotheses.

## 5 Analysis and discussion

### 5.1 Demographic profile

The synopsis of the demographics of the respondents is represented in Table 1.

**Table 1:** Demographic profile of the respondents (n = 432).

Characteristics and its category		Percentage (%)
Gender	Male	84.5
	Female	15.5
Age range	18–25 years	25.9
	26–40 years	68.8
	41–55 years	5.3
Highest academic degree achieved	Graduation	47.0
	Post-graduation	50.5
	PhD	2.5
Job-status	Jobseeker	27.1
	Public sector job	7.2
	Private-sector job	57.8
	Businessperson/self-employed	7.9
Professional experience	No experience	13.9
	03 months–06 months	13.0
	07 months–12 months	6.2
	01 year–05 years	37.3
	06 years–10 years	22.2
	11 years–20 years	5.1
	21 years–35 years or more	2.3

Source: Authors' Own Calculation.

The respondents’ demographic profile is being presented in Table 1. Among the respondents, most of the respondents are male (84.5 percent) and the female percentage is 15.5. A large proportion (68.6 percent) of the respondents are in the 26–40 years age range category and 18–25 years and 41–55 years category, there are 25.9 and 5.3 percent of respondents participated respectively. All the respondents have completed at least their bachelor level degree. In the job status section, Table 1 represents that, among the respondents, 27.1 percent were job seekers, 7.2 percent working in the public or government sector, the majority (57.8 percent) working in different private-sector jobs, and finally, 7.9 percent were doing their own business. In the professional experience section, 13.9 percent had no professional experience and other 86.1 percent of the participants had at least three months of professional experience. Different categories of experience are illustrated in Table 1.

**Figure 2:**
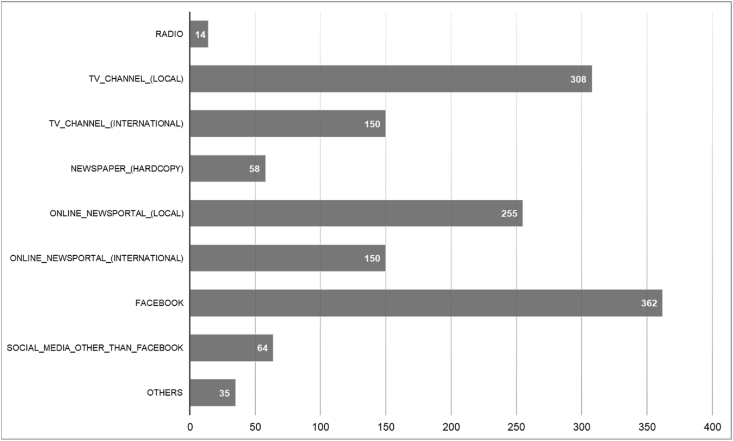
COVID-19 related information sources.

As this study is related to ‘COVID-19 phobia’ and the relationship of this phobia with career anxiety, thus it needs to know the sources of information where the respondents collecting information regarding COVID-19. Figure 2 illustrates the sources of collecting information in a bar diagram.

Figure 2 shows that, for information gathering purposes, Facebook remains the dominating platform, followed by local television channels, local online versions of the newspaper, and so on.

## 6 Results and discussion

A two-step approach suggested by Anderson and Gerbing (1988) was followed for the data analysis and hypothesis testing. Which means, first of all measurement model's validity and reliability has to be performed and then, structural model has to be confirmed.

### 6.1 The measurement model

A calculation of the measurement models included composite reliability to evaluate internal consistency, individual indicator reliability and average variance extracted (AVE) to evaluate convergent validity. By examining each loading for each block of indicators Convergent validity can be measured (Osman and Sentosa, 2013). Convergent validity is a scale's capability to load together as a single construct. Outer loadings values should be higher than 0.70, which indicates, sharing more variance with their respective latent variable than with error variance. Loadings ranging from 0.40 to 0.60 may also be acceptable in exploratory research although 0.70 has been considered as benchmark (Chin, 1998; Hulland, 1999). In this analysis, standardized indicator loadings are ranging from 0.58 to 0.88, which is within the prescribed acceptable range.

Variance Inflation Factor (VIF) quantifies the severity of collinearity among the indicators in a formative measurement model. The recommended value for VIF is less than 05 (Hair et al., 2014). The VIF values are ranging from 1.21 to 3.74 in this study, which is within the satisfactory range.

Internal consistency is typically calculated using Cronbach's α. Hair et al. (2014) suggested the Cronbach's α value should be higher than 0.60. All the values of Cronbach's α are in a satisfactory range depicted in Table 2 (ranging from 0.68 to 0.91).

**Table 2:** Reliability and validity assessment.

Factor/Constructs	Items	Loadings	VIF	Cronbach's α	Average Variance Extracted (AVE)	Composite Reliability (CR)	rho_A
Psychological factors	Psy1	0.71	1.86	0.79	0.48	0.85	0.80
	Psy2	0.75	1.71				
	Psy3	0.70	1.63				
	Psy4	0.70	1.28				
	Psy5	0.72	1.55				
	Psy6	0.58	1.21				
Social factors	Social1	0.62	1.44	0.72	0.46	0.81	0.75
	Social2	0.69	1.53				
	Social3	0.59	1.21				
	Social4	0.73	1.32				
	Social5	0.76	1.29				
Economic factors	Eco1	0.81	1.50	0.68	0.50	0.80	0.73
	Eco2	0.79	1.54				
	Eco3	0.60	1.74				
	Eco4	0.60	1.74				
Psycho-somatic factors	PsySom1	0.82	1.90	0.91	0.72	0.93	0.92
	PsySom2	0.83	3.00				
	PsySom3	0.88	3.74				
	PsySom4	0.86	2.36				
	PsySom5	0.86	2.76				
Career Anxiety	CA1	0.82	2.29	0.89	0.64	0.91	0.89
	CA2	0.78	1.90				
	CA3	0.82	2.08				
	CA4	0.85	2.56				
	CA5	0.84	2.57				
	CA6	0.69	1.44				

Source: Output from SmartPLS v3.2.9.

For Average Variance Extracted (AVE) and Composite Reliability (CR), Hair et al. (2019) recommended the threshold, 0.50 and 0.70 consecutively. Even though Malhotra and Dash (2016) argued that “*AVE is a more conservative measure than CR*,” so based on the value of CR alone, it can be concluded that the constructs are valid and reliable. The CR ranges between 0 and 1, higher values representing higher levels of reliability. For indicating the adequacy, values from 0.60 to 0.70 are acceptable in exploratory research, while in more advanced stages of research, values above 0.70 are viewed as satisfactory (Fornell and Larcker, 1981; Hair et al., 2017). It can be identified from the table-2 that, all the values for CRs are well above the threshold.

Recent studies show that Dijkstra-Henseler statistics (rho_A) is another indicator of reliability and validity. The value of the rho_A coefficient should be more than 0.60 (Dijkstra and Henseler, 2015). In this study, the values of rho_A are ranging from 0.73 to 0.92 which met the prescribed criteria.

Next, discriminant validity was assessed. The left part of Table 3 shows the correlations between the constructs (Fornell Larcker Criterion). According to (Hair et al., 2017), “*the logic of the Fornell-Larcker method is based on the idea that a construct shares more variance with its associated indicators than with any other construct*.” The square root of each AVE (shown on the diagonal) is greater than the related inter-construct correlations in the construct correlation matrix, indicating adequate discriminant validity for all of the reflective constructs (Fornell and Larcker, 1981; Henseler et al., 2009).

**Table 3:** Discriminant validity assessment.

	Fornell Larcker Criterion					Heterotrait-Monotrait Ratio (HTMT)				
	1	2	3	4	5	1	2	3	4	5
Career Anxiety	**0.80**									
Economic factor	0.51	**0.71**				0.61				
Psycho-somatic factors	0.39	0.50	**0.85**			0.41	0.66			
Psychological factors	0.45	0.34	0.32	**0.70**		0.51	0.46	0.38		
Social factor	0.43	0.45	0.40	0.50	**0.68**	0.50	0.63	0.46	0.66	

Note(s): Values on the diagonal (bold) are the square root of the AVE while the off-diagonals are correlations. Source: Output from SmartPLS v3.2.9.

Heterotrait- Monotrait (HTMT) ratio of correlation is another measure to evaluate discriminant validity (Henseler et al., 2015) which has high power in the point out validity issues in variance-based SEM. All the values of the HTMT for this study, are below the suggested 0.90 thresholds (Table 3, right part), indicating a related relationship between indicators and constructs (Henseler et al., 2015).

The measurement model assessment showed the reliability and validity of constructs met all the required criteria. Thus, based on the hypotheses developed for this study, the next section can be performed which represents the structural relationship.

### 6.2 The structural model

The results related to the hypotheses formulated for this study presented in Table 4. By applying a 5000-subsample bootstrapping test, the statistical significance of the path coefficient was estimated for this study. Chin (1998), pointed out a coefficient of determination (R^2^) of the endogenous latent variables is the crucial criterion for evaluating the structural model. For considering a moderate relationship the value of R^2^ must be above 0.2. The R^2^ value of endogenous construct (career anxiety) is 0.364 (adjusted value 0.358) which passes through the required minimum criteria (Hair et al., 2017).

**Table 4:** Hypotheses testing (Bootstrapping) and Endogenous Constructs Assessment.

Path coefficients and bootstrapping					
Hypotheses	Paths	Path Coefficient (β)	T Statistics (|O/STDEV|)	P Values	Significance
H1	Psychological factors - > Career Anxiety	0.241	5.183	0.000	Yes∗∗
H2	Social factor - > Career Anxiety	0.123	2.347	0.019	Yes∗
H3	Economic factor - > Career Anxiety	0.326	6.489	0.000	Yes∗∗
H4	Psycho-somatic factors - > Career Anxiety	0.094	2.09	0.037	Yes∗

Endogenous constructs assessment			
	R Square	R Square Adjusted	Q Square
Career Anxiety	0.364	0.358	0.223

[Notes: ∗p-value < 0.05. ∗∗p-value<.001]

[Note: T Statistics (|O/STDEV|) and P Values are computed through bootstrapping procedure with 432 cases and 5,000 samples.]

Regarding the measures of approximate adjustment of the model (Henseler et al., 2016), the value is obtained from the residual root mean square (Hu and Bentler, 1999), known as the SRMR. The SRMR value should be below 0.080 indicating a model has a goodness of fit (Hu and Bentler, 1999). In this study, SRMR = 0.075 is very close, but within the acceptable range.

For the predictive capacity of the model, the blindfolding procedure was applied for this study. The predictive relevance of the model was studied through the Stone-Geisser test (Q^2^) which exposed that the model is predictive (Q^2^ = 0.223) since Q^2^ > 0 and indicative of predictive relevance; 0.02, 0.15, 0.35 for a weak, moderate, and strong degree of predictive relevance suggested by Hair et al. (2017) and Chin (2010).

The result shown in Table 4 represents that all the four-hypothesis developed for this study are significant. Two hypotheses (H_1_ and H_3_) were supported at .001 significance level and the other two hypotheses (H_2_ and H_4_) were supported at .05 significance level. The *β* value, *t* Statistics, and *P*-value for each of the hypotheses are illustrated in Table 4.

The empirical results reveal that all the four factors of the ‘COVID-19 phobia’ scale (i.e., Psychological, Social, Economic, and Psycho-somatic) have a significant influence on career anxiety. Due to the spread of COVID-19, human psychology become heavily affected as because people tend to think about themselves and their family members as they may be affected by the virus. With this, the surge of career related anxiety getting multiplied. Similarly, as humans are a part of society, thus, any interruption in the regular social activity heavily impact on human mind. Due to the spread of COVID-19, people tend to think about their surroundings which hamper their regular career related thinking and trigger career anxiety. Again, for the economic factors, the regular flow of food supply and other livelihood items are getting hampered which also impacted on career related anxiety. Finally, the psycho-somatic factors, due to the pandemic, human are becoming anxious regarding their psychological health related issues (i.e., sleeping, stomach problem, chest pain, high blood pressure) which increases the anxiety level and make a fearful situation for the people's career related thoughts. Due to the second wave of the coronavirus spread and economic downturn, people from all around the world are losing jobs and are facing an uncertain future. Although some vaccines are giving some promising news but it will take a considerable amount of time to vaccinate all the people of the world. Thus, the empirical research and the research findings indicate that, because of the outbreak of coronavirus, ‘COVID-19 phobia’ developed into the mindset of people and that triggers career anxiety among the workforce which have a long-term negativity to them.

## 7 Conclusion and implication

Because of the COVID-19 outbreak, human psychology is affecting severely (Shammi et al., 2020) and novel breeds of anxiety disorders growing into the mindset of people all around the world. Relative younger generations are the worst sufferer in this pandemic as depression is rooting into their mindset and creating long-term negativity into their mentality (Mahmud et al., 2020). This study employed the ‘COVID-19 phobia’ scale to examine, whether the factors associated with ‘COVID-19 phobia’ have any influence on career anxiety or not. The empirical results reveal that all the four factors of the ‘COVID-19 phobia’ scale (i.e., Psychological, Social, Economic, and Psycho-somatic) have a direct and significant influence on career anxiety. Meaning, because of the COVID-19 outbreak, a special kind of phobia developed into the mindset of people, which is entirely new, and that phobia creating career anxiety among the current and future workforces' mindset. As the spread of the virus and the death toll continues, there is a possibility to witness a massive crackdown on human psychology all around the world and long-term adverse effects may shade the existing social structures in many folds. The study has both theoretical and practical implications. With the aid of this research theoretically new dimensions are being added on specific phobia under the Generalized Anxiety Disorder (GAD). On the other hand, practically, the policymakers and the concerned authorities will find some guidelines while designing a boost-up plan for the ‘lockdown generation’ (United Nations (UN), 2020b) and offer some distinct motivational packages (i.e., unemployment insurance, economic impact payments, economic stimulus relief) to flourish the potential of different age groups' workforce for the retrieval of world's economy.

## 8 Limitations and future research direction

For this study, the data was collected data in a state where all the countries of the world are maintaining social distancing to avoid a second wave of infection. It would be better to have a larger sample size and cross-cultural or multi-national study to generalize the concept. Moreover, this study collected data only from persons who have internet connectivity. The generalizability of the study result may not be possible because of this reason also. For future study, the model used for this study can be examined by applying some demographic variables as a moderator to analyze the model in a separate dimension.

## Declarations

### Author contribution statement

Md. Shahed Mahmud: Conceived and designed the experiments; Performed the experiments; Analyzed and interpreted the data; Contributed reagents, materials, analysis tools or data; Wrote the paper.

Md. Mahbubar Rahman: Analyzed and interpreted the data; Contributed reagents, materials, analysis tools or data; Wrote the paper.

Md. Masud-Ul-Hasan: Contributed reagents, materials, analysis tools or data; Wrote the paper.

Md. Azharul Islam: Conceived and designed the experiments; Performed the experiments; Wrote the paper.

### Funding statement

This research did not receive any specific grant from funding agencies in the public, commercial, or not-for-profit sectors.

### Data availability statement

Data will be made available on request.

### Declaration of interest statement

The authors declare no conflict of interest.

### Additional information

No additional information is available for this paper.

## Appendix A Supplementary data

3._Master_file_432data.sav 3._Master_file_432data.sav

1. Main Document 1. Main Document
