# Supplementary material for: Does ‘COVID-19 phobia’ stimulate career anxiety?: Experience from a developing country
Source: Heliyon. 2021 Mar 8;7(3):e06346. doi: 10.1016/j.heliyon.2021.e06346 (PMC8035488; doi:10.1016/j.heliyon.2021.e06346)
Supplement: 1. Main Document [file mmc2.docx]

**Appendix:**

*Measurement Scales*

| Psychological factors  (Arpaci et al., 2020) | Psy1 | The fear of coming down with coronavirus makes me very anxious. |
| --- | --- | --- |
|  | Psy2 | I am extremely afraid that someone in my family might become infected by the coronavirus. |
|  | Psy3 | News about coronavirus-related deaths causes me great anxiety. |
|  | Psy4 | Uncertainties surrounding coronavirus cause me enormous anxiety. |
|  | Psy5 | The pace that coronavirus has spread causes me great panic. |
|  | Psy6 | I argue passionately (or want to argue) with people I consider to be behaving irresponsibly in the face of coronavirus. |
| Social factors  (Arpaci et al., 2020) | Social1 | After the coronavirus pandemic, I feel extremely anxious when I see people coughing. |
|  | Social2 | After the coronavirus pandemic, I actively avoid people I see sneezing. |
|  | Social3 | Following the coronavirus pandemic, I have noticed that I spend extensive periods of time cleaning my hands. |
|  | Social4 | The fear of coming down with coronavirus seriously impedes my social relationships. |
|  | Social5 | I am unable to curb my anxiety of catching coronavirus from others. |
| Economic factors  (Arpaci et al., 2020) | Eco1 | The possibility of food supply shortage due to the coronavirus pandemic causes me anxiety. |
|  | Eco2 | The possibility of shortages in cleaning supplies due to the cornavirus pandemic causes me anxiety. |
|  | Eco3 | I stock food with the fear of coronavirus. |
|  | Eco4 | After the coronavirus pandemic, I do not feel relaxed unless I constantly check on my supplies at home. |
| Psycho-somatic factors  (Arpaci et al., 2020) | PsySom1 | I experience sleep problems out of the fear of coronavirus. |
|  | PsySom2 | I experience serious stomachaches out of the fear of coronavirus. |
|  | PsySom3 | I experience serious chest pain out of the fear of coronavirus. |
|  | PsySom4 | Coronavirus makes me so tense that I find myself unable to do the thing I previously had no problem doing. |
|  | PsySom5 | I experience tremors due to the fear of coronavirus. |
| Career Anxiety  (Tsai et al., 2017), and (Schmalbach et al., 2020) | CA1 | I worry about my career because of a potential economic recession due to the outbreak of COVID-19. |
|  | CA2 | I am always worrying about minor matters regarding my caerrer due to the outbreak of COVID-19 |
|  | CA3 | I worry about my career because of fierce competition in the job market due to the outbreak of COVID-19. |
|  | CA4 | I worry about my career and income due to the outbreak of COVID-19 pandemic. |
|  | CA5 | I worry about my career because of the increasing unemployment and job cut reported by the mass media for the reason of COVID-19. |
|  | CA6 | my colleagues or family members have already told me that I should not always worry that much about my career |
